# Thermal desorption effects on fragment ion production from multi-photon ionized uridine and selected analogues†

**DOI:** 10.1039/d1ra01873f

**Published:** 2021-06-09

**Authors:** J. Bocková, A. Rebelo, M. Ryszka, R. Pandey, D. Mészáros, P. Limão-Vieira, P. Papp, N. J. Mason, D. Townsend, K. L. Nixon, V. Vizcaino, J.-C. Poully, S. Eden

**Affiliations:** School of Physical Sciences, The Open University Walton Hall Milton Keynes MK7 6AA UK s.p.eden@open.ac.uk; Atomic and Molecular Collisions Laboratory, CEFITEC, Department of Physics, FCT – Universidade NOVA de Lisboa P-2829-516 Caparica Portugal; Department of Experimental Physics, Comenius University in Bratislava Mlynská dolina F2 84248 Bratislava Slovakia; School of Physical Sciences, Ingram Building, University of Kent Canterbury CT2 7NH UK; Institute of Photonics and Quantum Sciences, Heriot-Watt University Edinburgh EH14 4AS UK; Institute of Chemical Sciences, Heriot-Watt University Edinburgh EH14 4AS UK; School of Life, Health, and Chemical Sciences, The Open University Walton Hall Milton Keynes MK7 6AA UK; School of Sciences, University of Wolverhampton Wulfruna Street Wolverhampton WV1 1LY UK; CIMAP UMR 6252, CEA/CNRS/ENSICAEN/Université de Caen Normandie, GANIL Bd Becquerel BP 5133 14070 Caen France

## Abstract

Experiments on neutral gas-phase nucleosides are often complicated by thermal lability. Previous mass spectrometry studies of nucleosides have identified enhanced relative production of nucleobase ions (*e.g.* uracil^+^ from uridine) as a function of desorption temperature to be the critical indicator of thermal decomposition. On this basis, the present multi-photon ionization (MPI) experiments demonstrate that laser-based thermal desorption is effective for producing uridine, 5-methyluridine, and 2′-deoxyuridine targets without thermal decomposition. Our experiments also revealed one notable thermal dependence: the relative production of the sugar ion C_5_H_9_O_4_^+^ from intact uridine increased substantially with the desorption laser power and this only occurred at MPI wavelengths below 250 nm (full range studied 222–265 nm). We argue that this effect can only be rationalized plausibly in terms of changing populations of different isomers, tautomers, or conformers in the target as a function of the thermal desorption conditions. Furthermore, the wavelength threshold behavior of this thermally-sensitive MPI channel indicates a critical dependence on neutral excited state dynamics between the absorption of the first and second photons. The experimental results are complemented by density functional theory (DFT) optimizations of the lowest-energy structure of uridine and two further conformers distinguished by different orientations of the hydroxymethyl group on the sugar part of the molecule. The energies of the transitions states between these three conformers are low compared with the energy required for decomposition.

## Abstract 2

This work reveals the first experimental evidence supporting isomer-dependence in the radiation response of a nucleoside.

## Graphical abstract

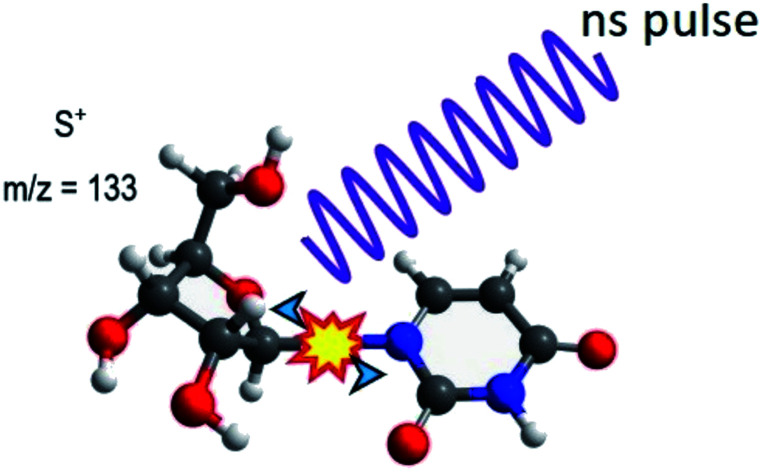

## 1. Introduction

**Fig. 1:**
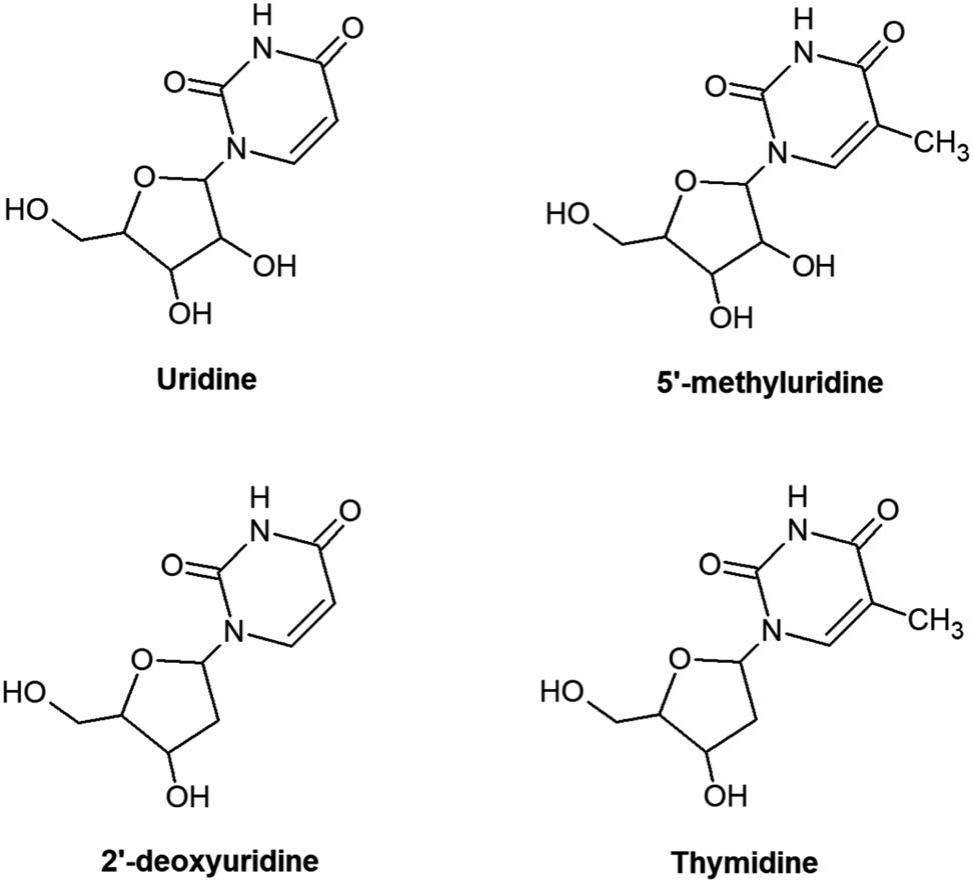
Chemical structures of the molecules studied in the present work. The diagrams are arranged such that the nucleosides with common sugar parts (ribose minus OH or deoxyribose minus OH) align horizontally, while those with common base parts (dehydrogenated uracil or dehydrogenated thymine) align vertically.

Understanding the radiation response of nucleic acids and their subunits can carry implications for the molecular origins of life and guide future innovations in radiotherapy and radioprotection. This rationale has inspired numerous experimental^1–6^ and theoretical^3,7–9^ studies of radiation-induced processes in neutral and charged targets ranging in complexity from gas-phase nucleobases to condensed hydrated RNA/DNA. Targeting relatively small sub-units in the gas phase generally affords the clearest physical–chemical interpretations, particularly *via* synergies between experiments and high-level calculations. However, it is often difficult to assess the relevance of such studies to more complex irradiated systems. A natural way to tackle this problem is to compare spectroscopic features and/or reaction products from sequentially larger molecules, *e.g.* nucleobases, then nucleosides, nucleotides, oligonucleotides, RNA/DNA strands and beyond.^10–12^ Electrospray ionization sources can give access to experimentally-viable gas-phase targets across a great range of complexities,^13–16^ but even the first step in this sequence presents a challenge for neutrals due to thermal lability.^17,18^ Accordingly, the experimental literature on neutral gas-phase nucleosides is sparse compared with nucleobases.^4^ This paper presents the first multi-photon ionization (MPI) experiments on the RNA nucleoside uridine and two analogues: 5-methyluridine and 2′-deoxyuridine (shown schematically in Fig. 1; we also tested thymidine but could not rule out some contribution of thermal decomposition in this specific target). To our knowledge, the only previous gas-phase experiments on these three neutral molecules have involved rotational spectroscopy, dissociative electron attachment, anion formation in collisions with electronegative atoms, or direct access to cationic states by electron impact, single photon absorption, or chemical ionization.^18–23^ This contribution demonstrates the efficacy of the laser-based thermal desorption technique developed by Greenwood and co-workers^4^ for producing gas-phase targets of these nucleosides without thermal decomposition, while separate thermally-dependent and wavelength-dependent effects in the MPI mass spectra of uridine are interpreted in terms of changing populations of isomers/tautomers/conformers with differences in electronic state energies and dynamics.

A number of previous studies have shown that it is possible to produce intact neutral pyrimidine-based nucleosides in the gas phase by heating in oven systems (with or without a carrier gas).^17,18,22^ However, most works did not report direct tests of target purity,^23–28^ and those that did either showed that some thermal decomposition was present in all the conditions applied^29^ or that pure targets could only be produced in narrow temperature ranges.^18–20^ This suggests that there is value in exploring alternative methods to produce gas-phase nucleosides with relatively high target density and without thermal decomposition. Only one paper has reported experiments on gas-phase uridine from a non-oven source: Peña *et al.*^19^ used a laser ablation system for their rotational spectroscopy study of uridine but significant populations of thermal reaction products were also present in the target. Indeed, it is not possible to avoid some contribution of decomposition in gas-phase targets produced by laser pluses impinging directly on a condensed sample.^10^ This is tolerable in various types of experiment which only require a high abundance of the intact molecule of interest (*i.e.* they are not significantly compromised by the presence of some other species in the target) but it presents a problem in dissociative ionization studies. Laser-based vaporization methods in which the beam does not interact directly with the condensed sample generally offer better prospects for avoiding any thermal decomposition. Laser-induced acoustic desorption (LIAD) is the best-known method of this kind; laser pulses striking one side of a metal foil result in desorption from a thin sample layer (≤μm-order thickness) on the other side *via* a combination of thermal and non-thermal processes.^30,31^ Laser-based thermal desorption^4,32,33–35^ differs from LIAD in that it applies a continuous wave (CW) laser to heat the foil and the desorption process is purely thermal. This relatively new technique (first reported in 2015 (ref. 4)) is understood to be amenable to studying thermally labile species for two main reasons. Firstly, the heating-up time is ≤ a few seconds due to the low thermal mass of the foil and the sample. Previous works have identified shorter heating-up times as favoring phase changes *versus* thermal decomposition.^36^ Secondly, condensed molecules remain hot for short periods compared with typical oven source applications. Molecules are removed from a small hot area of the foil quite quickly (*e.g.* within several tens of minutes) before the laser spot or the foil is moved, and a new small area of the condensed sample is heated. Furthermore, the time during which thermal decomposition can take place in the gas phase is kept to a minimum because molecules do not interact with any hot surfaces after sublimation and the condensed sample is mounted just a few mm from the ionizing laser beam (or an alternative probe).

Greenwood and co-workers have applied laser-based thermal desorption in three studies of gas-phase nucleosides^4,37,38^ (thymidine, adenosine, guanosine, and cytidine). These papers showed that a large proportion of the targets comprised intact nucleosides, but they did not present investigations of possible thermal decomposition as a function of desorption laser power. The only study of this kind for this vaporization method was Ghafur *et al.*'s^33^ demonstration that the ratios of fragment ion/parent ion signals from photoionized uracil (a much less thermally-labile molecule than any nucleoside) were independent of the desorption laser power. The only previous systematic study of the thermal decomposition of uridine^18^ revealed increased relative production of uracil^+^ (*m*/*z* 112) by single-photon ionization when the condensed sample was heated above 140 °C in an oven system. No other thermal dependences in relative fragment ion signals from uridine have been reported at lower temperatures. Similar findings from mass spectrometry experiments on other nucleosides^17,18,22^ have led to a general acceptance that enhanced relative production of nucleobase cations (typically referenced to the protonated nucleobase signal or to the total ion signal) can be treated as the first indicator of thermal decomposition. This provided the test for thermal decomposition in the present MPI experiments.

Decomposition is not the only way that thermal desorption conditions can modify a gas-phase molecular target. Typically, some changes in the isomeric form of a biomolecule (using the term isomer in its most general sense here, including different tautomeric forms and conformers) on the scale of a nucleoside can be anticipated at temperatures which are too low for significant decomposition. The present DFT calculations provide the first test of how the barriers for selected isomeric transitions of uridine compare with the minimum energy required for thermal decomposition. Previous theoretical calculations have identified a number of different stable isomers of uridine^9,19,39^ and 2′-deoxyuridine.^9^ Although only one gas-phase uridine structure has been identified experimentally to date,^19^ the authors did not rule out the possible presence of additional isomers in their laser-ablated target. Furthermore, isomer populations in one experimental gas-phase target may be different to another depending on temperature and desorption method. The structures of nucleosides within nucleic acids can also be modified during cellular processes, most obviously replication. Therefore, characterizing the effects of initial structure is important for any future attempts to link insights from gas-phase studies of irradiated nucleosides to biological radiosensitivity. Delchev's^9^ time-dependent DFT study revealed significant changes in the vertical energies and oscillator strengths of the lowest-lying singlet excited states of uridine and of 2′-deoxyuridine in different isomeric forms, including the ordering of the nπ* and ππ* states localized on the aromatic ring of the base part. However, to our knowledge, no other studies have considered the possible differences in the radiation response of different isomers of the nucleosides studied here. Fragment ion production by nanosecond-timescale MPI can be sensitive to neutral excited state dynamics initiated by the absorption of the first photon^1^ so the present experiments as a function of thermal desorption conditions can yield new clues into how isomeric form affects electronic excited state processes in these important biomolecules.

## 2. Experimental methods

**Fig. 2:**
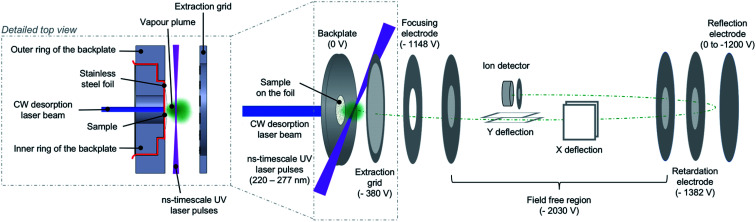
Scheme of the MPI-TOF experiment in laser-based thermal desorption mode. The desorption source is shown in greater detail.

The present measurements were carried out using a multi-photon ionization – time-of-flight (MPI-TOF) mass spectrometry set-up at the Open University (OU). This has been described previously for experiments on molecules and clusters in supersonic beams.^1,2,40–42^ With the exception of one measurement which is mentioned only briefly in this paper, the molecular targets in the present experiments were produced under vacuum by laser-based thermal desorption. Fig. 2 is a schematic representation of the MPI-TOF experiment in laser-based thermal desorption mode. MPI was achieved using partially-focused UV laser pulses (Continuum Powerlight II 8000 – Sirah Cobra-Stretch, pulse width 7 ns) in the wavelength range 222–265 nm at an approximate fluence of 5 × 10^6^ W cm^−2^. The mass spectrometer is a reflectron system built by KORE Technology interfaced with a Fast ComTec P7887 time-to-digital conversion (TDC) card.

The OU laser-based thermal desorption source is a simple adaptation of systems developed by Greenwood, Townsend and co-workers at Queen's University Belfast^4,37,38^ and Heriot-Watt University.^33–35^ It has been exploited in previous published work on uracil^33^ and 5-fluorouracil^32^ but its specifics are described here for the first time. The backplate of the mass spectrometer has been modified such that a piece of stainless-steel foil (thickness 10 μm) can be gripped between an inner ring and an outer ring as shown in the detailed top view of Fig. 2. Importantly, there is a direct line of sight through the hole (diameter 8 mm) in the inner ring that allows a CW laser beam to hit the reverse side of the foil (the side facing away from the ion extraction volume). The front side of the foil (where the sample is deposited) and the outer ring present a flat surface so the modification of the backplate does not disrupt the ion extraction field.

The backplate is removed from the vacuum chamber and mounted horizontally for sample preparation. This involves placing 30–40 mg of sample powder (from Sigma-Aldrich with stated purity of ≥99% for uridine, 2′-deoxyuridine and thymidine, and ≥97% for 5-methyluridine) onto the foil, adding a few drops of methanol (Fisher Scientific, stated purity ≥ 99.8%), spreading using a spatula, and then leaving to allow the methanol to evaporate in air (1 bar, 296 K). The resulting layer of adhered nucleoside has a thickness of order 100 μm. The backplate is then re-installed in the chamber, which is then pumped down.

During experiments, the reverse side of the foil is irradiated using a CW diode laser (Eksma Optics DLM-445-1000, 445 nm, variable output power up to 1 W) and the beam enters the vacuum chamber *via* a standard borosilicate glass viewport (∼92% transmission). The CW laser is mounted on a 3-way translator so the spot can be directed onto different parts of the foil, allowing more measurements per sample preparation. The spot has an elliptical shape (major axis 4.5 mm and minor axis 1 mm) and it was unfocused in the present experiments. The CW laser heats the foil and hence heats the condensed sample indirectly to cause thermal desorption. Some desorbed molecules are multi-photon ionized by the UV laser pulses which pass 1–2 mm in front of the condensed sample. A series of measurements were performed *ex situ* using an adhered thermocouple (Omega SA3-K) to determine the temperature of the hottest part of the foil to within ±5 °C as a function of the CW laser power.^43^

## 3. DFT optimizations of selected uridine conformers and dissociation products

The base part of uridine is dehydrogenated uracil (111 amu) and the sugar part is ribose minus OH (133 amu). For brevity, we will refer to a dehydrogenated nucleobase as (B–H), and to the sugar part of a nucleoside as S. This paper focuses on the fragments produced by cleavage of the glycosidic bond between (B–H) and S with or without the transfer of one or two hydrogen atoms. Therefore, the hydrogen atoms that interact with both parts of uridine and those in the immediate vicinity of the glycosidic bond are particularly relevant in the present discussion.

**Fig. 3:**
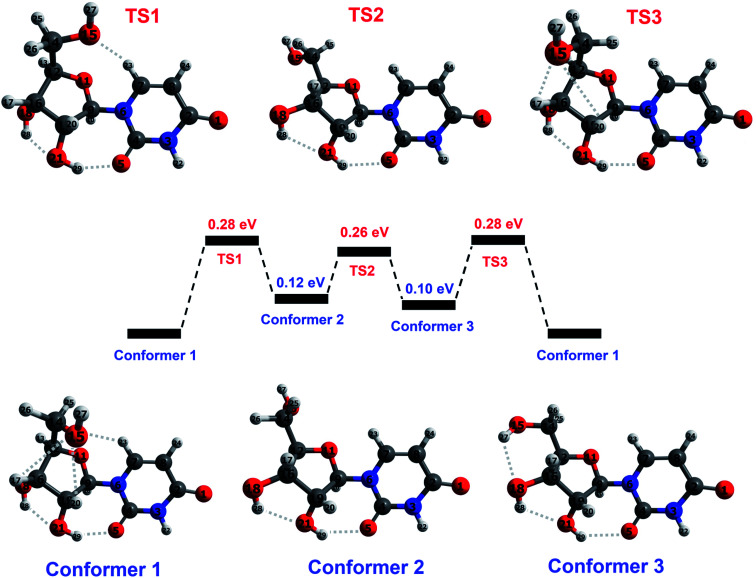
Illustration of the reaction pathway concerning hydroxymethyl group rotation in ground-state uridine, calculated at M05/aug-cc-pVDZ level. Conformer 1 corresponds to the lowest-energy structure identified by Peña *et al.*^19^ on the basis of *ab initio* calculations and rotational spectroscopy experiments. Conformer 2 corresponds to the lowest-energy structure from DFT calculations by Delchev^9^ and by So and Alavi.^39^ Conformer 3 has not been reported previously. Transition states between each conformer correspond to a maximum in the potential energy along the reaction coordinate. Hydrogen bonds are indicated by dotted lines and atoms are colored as follows: blue for nitrogen, dark gray for carbon, red for oxygen, and light gray for hydrogen. Selected parameters of the optimized conformers and transition states are given in Table 1.

Peña *et al.*^19^ performed an exhaustive conformational search using force field and semi-empirical methods and then carried out *ab initio* optimizations of the five lowest-energy structures. The resultant lowest-energy optimized structure showed excellent agreement with their rotational spectroscopy measurements on a target produced by laser ablation followed by cooling in a supersonic expansion. We performed DFT optimizations (M05/aug-cc-pVDZ and wB97XD/aug-cc-pVDZ – see the ESI† for details on the calculations including the functional/basis set choices) of Peña *et al.*'s^19^ structure in order to establish the starting point for our subsequent calculations. The structure (conformer 1 in Fig. 3) is characterized by five cooperative hydrogen bonds; their distances and three significant intramolecular angles are provided in Table 1. The similarity of our results with Peña *et al.*'s higher-level calculations indicates that the present level of theory and frameworks are suitable for describing the structure of isolated neutral uridine. Furthermore, it is reassuring that the two combinations of functional and basis set applied here give mutually consistent energies, bond distances, and angles for all the conformers and transition states considered in this work (maximum differences 0.05 eV, 0.14 Å, and 5°).

**Table 1:** Relative energies, selected angles, and hydrogen-bond distances in the three conformers and transitions states in Fig. 3. Note that the key differences between the structures lie in the orientation of the hydroxymethyl group, as defined by the C16–C12–C14–O15 angle and (somewhat less critically) by the O18–C16–C12–C14 angle. This motion of the hydroxymethyl group is accompanied by the formation and/or breaking of hydrogen bonds involving the O15 atom. The present calculations were performed at two levels and comparisons are drawn with previous calculated parameters, where available

Present DFT level/previous work	Conformer 1 (C1)				TS1 (C1 to C2)		Conformer 2 (C2)		TS2 (C2 to C3)		Conformer 3 (C3)		TS3 (C3 to C1)	
	M05	WB97XD	Peña^19^		M05	WB97 XD	M05	WB97XD	M05	WB97XD	M05	WB97XD	M05	WB97XD
Energy (eV) re. conformer 1	0	0	0		0.28	0.28	0.12	0.17	0.26	0.31	0.10	0.13	0.28	0.27

**Angles** a **(°)**														
C16–C12–C14–O15	55	55		53	118	116	187	187	243	247	289	290	350	350
O18–C16–C12–C14	147	150		153	151	155	80	81	77	79	77	78	153	158
C7–N6–C9–O11	−173	−174		−179	−174	−177	−176	−176	−174	−175	−174	−175	−179	−179

**H-bond distances** b **(Å)**														
H29⋯O5	1.92	1.91		1.93	1.86	1.83	2.13	2.11	2.22	2.19	2.29	2.21	1.88	1.90
H28⋯O21	2.05	2.03		2.02	2.09	2.09	2.01	2.01	2.07	2.05	2.08	2.07	2.12	2.10
H20⋯O15	2.57	2.50		2.41	—	—	—	—	—	—	—	—	2.94	2.80
H23⋯O15	2.94	2.84		2.97	2.36	2.34	—	—	—	—	—	—	—	
H27⋯O18	—	—		c	—	—	—	—	—	—	2.38	2.32	—	
H27⋯O15	2.86	2.87		c	—	—	—	—	—	—	—	—	2.30	2.30

a These three angles are highlighted in accordance with Peña *et al.*'s^19^ work.

b 3.00 Å is treated here as the maximum distance for an interaction to be classed as CH⋯O or OH⋯O bonding.

c These distances were not provided in Peña *et al.*'s^19^ paper. Their diagram of the conformer makes it obvious that the H27 atom is too distant from the O18 atom for any significant interaction, whereas it appears to be sufficiently close to the O15 atom for hydrogen bonding.

Earlier DFT calculations by Delchev^9^ and by So and Alavi^39^ identified a different lowest-energy structure^9,39^ of uridine, corresponding to conformer 2 in Fig. 3. Delchev^9^ presented another five uridine isomers with higher energies but these did not include conformer 1. As shown in Table 1, conformer 1 is more stable than conformer 2 by 0.12–0.17 eV in the present optimizations. The two conformers have very similar structures aside from the orientation of the hydroxymethyl group (CH_2_OH) on the sugar. The hydroxymethyl group rotation required for the conformers to approach each other must overcome barriers associated with interactions of the O15 atom with the C7–H23 and C19–H20 bonds. Further rotation about the C12–C14 bond and optimization brings the molecule into a stable configuration (conformer 3) that has not been reported before. Transition states for interconversion between these three conformers are shown schematically in Fig. 3 and summarized in Table 1. The calculated barriers for conversion from conformer 1 into conformer 2 or conformer 3 are 0.27–0.28 eV. Transitions between conformers 2 and 3, or from either into conformer 1 require between 0.11 and 0.18 eV. These energies are low compared with the previously-reported barriers for isomeric transitions of neutral uridine, notably ∼0.75 eV rotation of the base ring plane about the glycosidic bond axis (C7–N6–C9–O11)^44^ and ≥1.25 eV for a selection of intramolecular proton transfer reactions.^9^

Possible thermal decomposition products of uridine were investigated as follows. Starting from conformer 1, we split the molecule at the glycosidic bond with the transfer of up to two hydrogens from the base part of the molecule to the sugar part or *vice versa*, and then optimized the resultant structures at infinite separation. We then compared the summed energy of each product-pair with the energy of conformer 1. This was repeated for hydrogen transfer between all of the possible sites on either side of the glycosidic bond and many of the transfers resulted in multiple stable structures. The summed energy of the lowest-energy pair (B and S–H) was 0.63 eV higher than conformer 1 (see the ESI† for more details). Hence dissociation requires at least this energy, which is more than double any of the barriers between the three conformers considered here. This confirms that thermally-driven conformational changes in gas-phase uridine will occur at temperatures which are too low for decomposition to take place.

## 4. Experimental results and discussion

Our first MPI experiments on uridine (data not shown) were carried out using a resistively-heated nozzle system for seeding molecules in supersonic argon or helium beams.^1,2,40–42^ It was necessary to heat the powder to 252 °C in a helium flow to obtain reasonable signals but this caused thermal decomposition. The mass spectrum was dominated by numerous small fragment ions, no S^+^ or (S–H)^+^ ions were detected, and the sample remaining in the nozzle after the experiment was discolored. As expected for ionization measurements of significantly thermally-decomposed nucleosides,^17,18,22^ the B^+^ signal (the uracil^+^ radical cation) dominated over the BH^+^ signal (protonated uracil). This confirmed the necessity of using a different vaporization system for the present experiments and led to our adoption of laser-based thermal desorption.

**Fig. 4:**
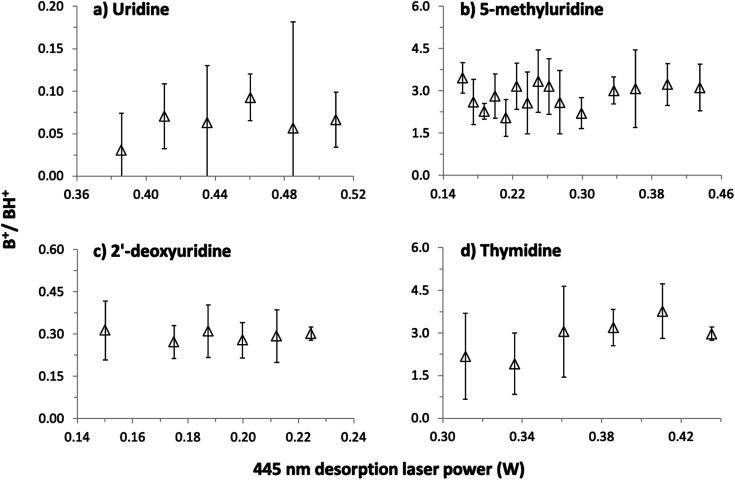
Ratios of nucleobase^+^ (B^+^, *e.g. m*/*z* 112 for uracil^+^) over protonated nucleobase (BH^+^, *e.g. m*/*z* 113 for protonated uracil) production by 225 nm MPI of (a) uridine, C_9_H_12_N_2_O_6_, (b) 5-methyluridine, C_10_H_14_N_2_O_6_, (c) 2′-deoxyuridine, C_9_H_12_N_2_O_5_, and (d) thymidine, C_10_H_14_N_2_O_5_, as a function of the desorption laser power. The desorption laser power ranges correspond to foil temperature ranges of 133–167 °C for uridine, 72–151 °C for 5-methyluridine, 67–93 °C for 2′-deoxyuridine, and 119–151 °C for thymidine.

The possibility of thermal decomposition of uridine, 5-methyluridine, 2′-deoxyuridine, and thymidine produced by laser-based thermal desorption was investigated here by measuring MPI mass spectra as a function of the desorption laser power (and hence foil temperature). As noted in Section 1, no previous studies^17,18,22,45^ have reported thermal effects on the mass spectra of nucleosides below the threshold for enhanced relative production of nucleobase ions (B^+^). The photon energies and fluences used in the present experiments produce strong B^+^ signals by 2-photon ionization of gas-phase uracil^1^ and thymine,^42^ whereas we can only speculate about the possible efficiency of multi-photon ionizing (S–H) neutrals. Therefore, we have followed Levola *et al.*^18^ in monitoring the B^+^/BH^+^ ratio to look for evidence of thermal decomposition. The Fig. 4 data was recorded by 225 nm MPI and large statistical errors on some of the ratios are due to weak B^+^ signals, most notably from uridine. Notwithstanding the statistical errors, the absence of any systematic increase in the B^+^/BH^+^ ratios in panels (a)–(c) indicates that uridine, 5-methyluridine, and 2′-deoxyuridine did not undergo thermal decomposition in the investigated laser power ranges. Only the thymidine results suggest a weak increase in the B^+^/BH^+^ ratio with the desorption laser power. This is consistent with previous reports of thymidine being more thermally labile than uridine.^18,22^

**Fig. 5:**
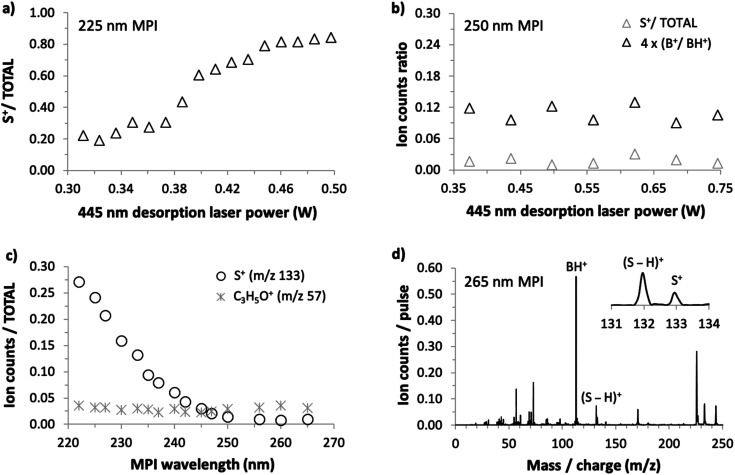
(a) Desorption laser power dependence of S^+^/total ion production by 225 nm MPI of uridine (foil temperature range 119–165 °C). (b) Desorption laser power dependence of S^+^/total ion production and B^+^/BH^+^ production by 250 nm MPI of uridine (foil temperature range 136–218 °C). (c) MPI wavelength dependence of the S^+^ and C_3_H_5_O^+^ ion signals/total ion production from uridine (desorption laser power 0.41 W, foil temperature 144 °C). (d) 265 nm MPI mass spectrum of uridine (desorption laser power 0.41 W, foil temperature 144 °C).

Additional tests involved acquiring MPI mass spectra of all four molecules under conditions matching the Fig. 4 experiments before and after heating the foil with a desorption laser power of 1 W for 150 s. The B^+^/BH^+^ ratios from uridine and 5-methyluridine were unchanged, indicating that the Fig. 4 and 5 experiments on these nucleosides were performed far from the threshold for permanent damage. However, the period of intense laser heating raised the B^+^/BH^+^ ratios from 2′-deoxyuridine and thymidine. This suggests that the additional OH group on the sugar part of uridine and 5-methyluridine has a stabilizing effect with respect to thermal decomposition. Note that characterizing and understanding the thermal stability of different DNA/RNA subunits has relevance beyond the practicalities of producing gas-phase experimental targets. The low-lying singlet electronic excited states of gas-phase nucleosides feature efficient and rapid (*e.g.* ∼100 fs for thymidine^37^) internal conversion pathways to the electronic ground state. Therefore, much of the dissociation of isolated nucleosides following electronic excitation can be expected to take place in high vibrational levels of the electronic ground state, enabling analogies to be drawn with thermal decomposition experiments.

As we could not obtain convincing evidence for achieving laser-based thermal desorption of thymidine without some thermal decomposition, this molecule is not discussed further here. Examples of our 225 nm MPI mass spectra of uridine, 5-methyluridine, and 2′-deoxyuridine are presented in the ESI.† All the prominent ions from uridine have been assigned in previous electron impact ionization and single photon ionization studies.^18,24,25,28,45^ The remainder of this paper focuses on the production of S^+^ (*m*/*z* 133, C_5_H_9_O_4_^+^) from uridine. This stands out because it is the only ion from intact uridine whose relative yield depends on the desorption laser power. Fig. 5(a) shows that S^+^ production by 225 nm MPI of uridine increases as a proportion of the total ion signal with increasing desorption laser power. We observed no equivalent thermal desorption dependence of the relative production of S^+^ by 225 nm MPI of 5-methyluridine, which shares the same sugar part as uridine. Hence, the base part of uridine appears to be critically involved in this thermal desorption effect. As the lowest-energy electronic transitions of nucleosides occur between molecular orbitals that are localized on the base part,^9,37^ this provides an early hint that neutral electronic excited state dynamics might play a role in the thermal desorption dependence of S^+^ production from uridine shown in Fig. 5(a). We will develop this idea in the next paragraphs with reference to the sensitivity of this fragment ion channel to MPI wavelength (drawing on all four panels of Fig. 5).

S^+^ ions have been observed and assigned previously in experiments that involved direct ionization from uridine's electronic ground state.^18,22,24,25,28,45^ Significantly, however, Levola *et al.*'s^18^ study of thermal-desorption driven effects on the single photon ionization mass spectra of uridine did not identify any dependence of the relative signal of S^+^ on the sublimation temperature. The thermal desorption dependence seen in Fig. 5(a) is new and, furthermore, it depends critically on MPI wavelength. Fig. 5(b) shows that the relative production of S^+^ by 250 nm MPI of uridine is independent of the desorption laser power. The desorption power range is greater than in Fig. 4(a) so the B^+^/BH^+^ ratio is also shown in Fig. 5(b) to confirm the absence of thermal decomposition of uridine in these conditions.

Fig. 5(c) shows the dependence of S^+^/total ion production from uridine on MPI wavelength from 222 to 265 nm. The ratio increases steadily with falling wavelength below a threshold of about 250 nm. Wavelength threshold behavior of this kind in our multi-photon ionization experiments indicates a critical energy threshold in a neutral electronic excited state. If the energy threshold corresponds to an isomeric transition or a dissociation then this is likely to modify fragment ion production by MPI.^1,42^ Ionic state thresholds do not manifest themselves as distinct wavelength thresholds in our experiments because any MPI laser fluence that produces a reasonably high ion signal involves several different orders of photon absorption within a given measurement (typically 2-photon absorption and 3-photon absorption dominate, while 4-photon absorption contributes relatively weakly^1^). To provide a further indication that the present wavelength threshold behavior of the S^+^ signal is not related to an ionic state threshold, Fig. 5(c) also shows the corresponding wavelength dependence for the C_3_H_5_O^+^ fragment signal. Ptasińska *et al.*'s^22^ electron impact ionization measurements revealed that C_3_H_5_O^+^ has a similar appearance energy (10.20 ± 0.05 eV) as S^+^ (10.39 ± 0.15 eV) but Fig. 5(c) shows that its relative signal does not change significantly with MPI wavelength.

Fig. 5(d) shows that S^+^ production is small but not negligible at the longest wavelength studied here. Taken together, the results in Fig. 5 suggest that two different processes are responsible for producing S^+^ ions from uridine in the present experiments: (i) a process activated at MPI wavelengths below 250 nm that is sensitive to desorption laser power and (ii) a process that is insensitive to MPI wavelength and to desorption laser power. It is natural to attribute process (ii) to the same mechanism as S^+^ production in electron impact ionization^22^ and single photon ionization experiments,^18^ which have shown no dependence on thermal desorption. Neither the neutral electronic excited states accessed en route to ionization (which naturally depend on wavelength) nor the thermal desorption conditions have significant effects on this dissociation process. The following discussion considers the possible causes of the MPI wavelength threshold behavior and the thermal desorption dependence of process (i).

As thermal decomposition can be discounted, the desorption laser power dependence of S^+^ production from uridine in Fig. 5(a) is most likely to be due to changes in the populations of different isomers in the target. There are numerous examples in the literature of isomeric effects on electronic excited state dynamics^9^ and on the relative production of fragment ions in mass spectrometry experiments.^46^ Therefore, the plausibility of this attribution depends primarily on the idea that the relative populations of different uridine isomers can change significantly as a function of the desorption laser power (and hence foil temperature) in the present experiments. The calculations in Section 3 show that conformational isomeric transitions of uridine can occur at much lower temperatures than decomposition, and a range of higher barriers have been reported for different isomeric transitions in the literature.^9,44^ Therefore, we expect the changing desorption laser power in the Fig. 5(a) experiments to modify the relative populations of different uridine conformational isomers, and possibly also tautomers, in the gas-phase target. More generally, there are numerous precedents in the literature for different sublimation temperatures leading to different gas-phase isomer populations.^47–49^

The paragraphs above argue that the thermal desorption dependence of S^+^ production from multi-photon ionized uridine *via* what we have called process (i) can be traced to changes in isomeric form prior to MPI. Furthermore, process (i) demonstrates wavelength threshold behavior and this is indicative of a critical mechanism in an electronic excited state. These two points can be viewed as being inter-related when we consider that vibronic excitation is an excellent tool for changing molecular structure. Delchev's time-dependent DFT calculations^9^ provide an intriguing clue into how this might account for the present MPI results. His potential energy surface (PES) calculations showed that internal conversion between *S*_1_(nπ*) or *S*_2_(ππ*) states and relatively dark charge transfer (CT) states of nπ* character can dramatically reduce the energy required for isomeric transitions in electronically-excited uridine. Hence, these CT states can act as bridges for structural changes in uridine following excitation to the bright *S*_2_(ππ*) state in the present experiments. The calculated vertical excitation energies of the CT states vary significantly (4.801–5.822 eV) in the different uridine isomers considered by Delchev.^9^ This variation suggests that the initial isomeric form of the ground-state molecule is likely to have a significant effect on the excitation energy required for access to a CT state *via* a conical intersection (CI). Therefore, we speculate that structural changes in electronically-excited uridine *via* CT states can enhance S^+^ production in the present MPI experiments and that the lowest-lying CI for internal conversion from *S*_2_ into a relevant CT state requires <250 nm (>5.0 eV) photoabsorption. In this case, increasing the photon energy beyond this threshold could enable access to CT states from more initial isomeric forms of uridine in the target and/or could access parts of the *S*_2_ PES that relax more readily to a relevant CI. The desorption power dependence could be explained by increasing the relative populations of isomers that give access to CT states at lower photon energies. Further work is required to test this idea and, if it is correct, to identify the specific isomers and CIs involved.

## 5. Conclusions

This work provides the first demonstration that laser-based thermal desorption can be used to produce gas-phase nucleoside targets without any thermal decomposition. This was achieved unambiguously for uridine, 5-methyluridine, and 2′-deoxyuridine, but not for thymidine. Moreover, the experiments on uridine show that desorption laser power dramatically affects the MPI production of sugar-unit ions (*m*/*z* 133) *via* a critical process with a threshold energy of 5.0 eV in the neutral electronically-excited molecule. This dependence on thermal desorption conditions is understood in terms of changing isomeric populations in the target, and the present DFT calculations show that isomeric transitions of uridine will occur at markedly lower temperatures than decomposition. Therefore, although the key dynamics are yet to be identified, this paper's most significant contribution is the first experimental evidence supporting isomeric effects on the radiation response of uridine. To our knowledge, this is the first experimental result of this kind for any gas-phase nucleoside. It highlights the importance of developing methods to produce neutral gas-phase nucleoside targets that are pure in terms of structure as well as in terms of eliminating thermal decomposition.

## Data availability statement

The data that support the findings of this study are available from the corresponding author upon reasonable request.

## Conflicts of interest

There are no conflicts to declare.

## Supplementary Material

RA-011-D1RA01873F-s001
