# Supplementary material for: Thermal desorption effects on fragment ion production from multi-photon ionized uridine and selected analogues
Source: RSC Adv. 2021 Jun 9;11(34):20612–21. doi: 10.1039/d1ra01873f (PMC9033967; doi:10.1039/d1ra01873f)
Supplement: RA-011-D1RA01873F-s001 [file RA-011-D1RA01873F-s001.pdf]

Electronic Supplementary Material (ESI) for RSC Advances.
This journal is © The Royal Society of Chemistry 2021

Thermal desorption effects on fragment ion production from multi-photon ionized uridine and selected analogues

J. Bocková[1], A. Rebelo[1,2], M. Ryszka[1], R. Pandey[1], D. Mészáros[3], P. Limão-Vieira[2], P. Papp[3], N. J. Mason[1,4], D. Townsend[5,6], K. L. Nixon[7,8], V. Vizcaino[9], J.-C. Poully[9], and S. Eden[1]*

[1] School of Physical Sciences, The Open University, Walton Hall, Milton Keynes, MK7 6AA, UK

[2] Atomic and Molecular Collisions Laboratory, CEFITEC, Department of Physics, FCT - Universidade NOVA de Lisboa, P-2829-516 Caparica, Portugal

[3] Department of Experimental Physics, Comenius University in Bratislava, Mlynská dolina F2, 84248 Bratislava, Slovakia

[4] School of Physical Sciences, Ingram Building, University of Kent, Canterbury, CT2 7NH, UK

[5] Institute of Photonics and Quantum Sciences, Heriot-Watt University, Edinburgh EH14 4AS, UK

[6] Institute of Chemical Sciences, Heriot-Watt University, Edinburgh EH14 4AS, UK

[7] School of Life, Health, and Chemical Sciences, The Open University, Walton Hall, Milton Keynes, MK7 6AA, UK

[8] School of Sciences, University of Wolverhampton, Wulfruna Street, Wolverhampton, WV1 1LY, UK

[9] CIMAP UMR 6252, CEA/CNRS/ENSICAEN/Université de Caen Normandie, GANIL, Bd Becquerel, BP 5133, 14070 Caen, France

* Corresponding author: s.p.eden@open.ac.uk

Electronic Supplementary Information

S1. DFT optimizations of selected uridine isomers and possible pairs of dissociation products

As a starting point for our subsequent calculations of neutral ground-state energy barriers for isomeric transitions as well as of the optimized structures and energies of dissociation product pairs, we ran DFT optimizations of structures with input geometries close to those of the lowest-energy isomers of uridine that had been reported in the literature[1–3]. All the energies reported in this work were calculated at 0 K and have not been corrected for the vibrational zero-point energy. We checked for the absence of negative frequencies to ensure that the optimized structures are indeed energy minima and not saddle points. The Minessota functional M05[4] and the Dunning's double zeta basis set[5] augmented with diffuse functions (M05/aug-cc-pVDZ) were applied in all calculations. The combination of this functional with this basis set shows good performance in estimating atomization energies and heats of formation[6–8]. The identification of the three transition states in Fig. 3 and Table 1 began with a non-optimized potential energy scan as a function of dihedral angle C16-C12-C14-O15, keeping all other structural parameters of conformer 1 frozen. For each of the local maxima in this scan, a B97D/6-31G*/W06 geometry optimization was performed with the C16-C12-C14-O15 angle kept frozen. Each optimized geometry with one negative vibrational frequency was then re-optimized with the same method but all geometrical

variables were activated for optimization together with a request to find the transition state rather than a local minimum. Finally, the optimization of the transition states together with the known conformers 1-3 were repeated at wB97XD/aug-cc-pVDZ level, in line with Sherill and co-workers' recommendation[9], and at M05/aug-cc-pVDZ level. The wB97XD and M05 functionals describe well both non-covalent interactions and isomerization energies, which are crucial in the present theoretical investigation. In particular, Mardirossian and Head-Gordon[10] reported them to be among the best functionals to predict the isomerization energies, thermochemistry, and barrier heights of non-covalently bound organic molecules and biomolecules in 84 benchmark tests. A restricted formalism was employed for closed-shell species whereas open-shell species (radicals) were described by unrestricted wavefunctions. The spin contamination of radicals was monitored to avoid including higher spin states in the wavefunction. The maximum spin operator value was 0.7506, meaning that the wavefunctions are mostly free from contamination and hence suitable to use in the calculations. All the calculations were performed using the default algorithms of Gaussian09[11].

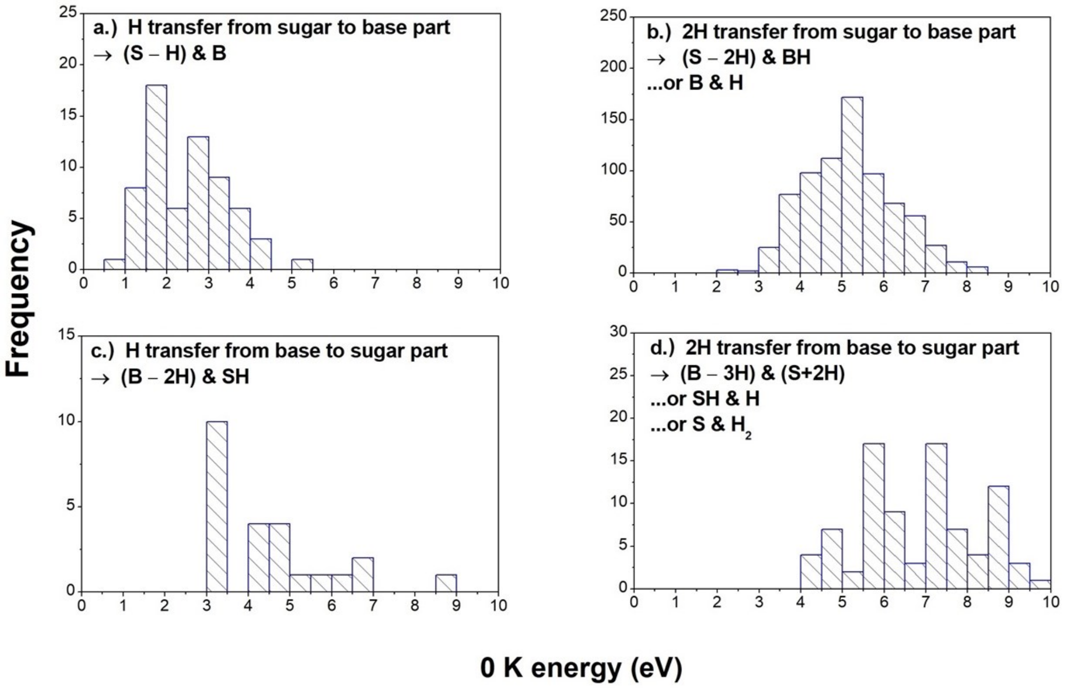

Fig. S1: Histograms showing energy differences between conformer 1 of uridine and the summed energies of optimized product-pairs that can be produced by glycosidic bond cleavage of uridine. The histograms are grouped according to the number of hydrogens transferred from the molecule's sugar part (S, 133 amu) to its base part ((B – H), 111 amu) or vice versa. The dissociation products are noted on the panels using the nomenclature described in the text.

Fig. S1 shows that by-far the lowest-energy calculated product-pairs of glycosidic bond cleavage are produced by single hydrogen transfer from the sugar to the base part of uridine. The resulting lowest-energy optimized B and (S – H) pair is shown schematically in Fig. S2(a) and has 0.63 eV greater energy than conformer 1, as noted in Section 3. Fig. S1 shows multiple energies corresponding to

product-pairs whose optimizations began with different possible hydrogen transfers during glycosidic bond cleavage. By contrast, only a single optimization of the S and (B – H) product-pair was carried out with the assumption that these are produced by a simple cleavage of the glycosidic bond without any reciprocal hydrogen transfers. The resultant optimized S and (B – H) pair has 3.61 eV greater energy than conformer 1 and is shown schematically in Fig. S2(b). This large minimum energy difference appears to be broadly consistent with the experimental evidence in Section 4 that the observed m/z 133 ions are not traced to thermal decomposition.

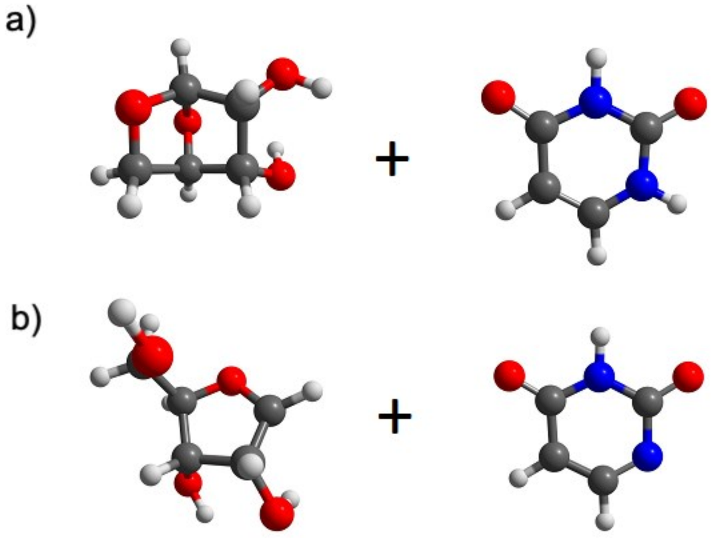

Fig. S2: (a) The lowest-energy optimized product-pair following glycosidic bond cleavage of *conformer 1* with single hydrogen transfer from the sugar to the base parts of uridine (i.e. giving uracil and an S – H isomer). (b) The optimized product-pair following glycosidic bond cleavage of *conformer 1* without any hydrogen transfer between the sugar and base parts (i.e. giving dehydrogenated uracil and an S isomer).

S2. MPI mass spectra of uridine, 5-methyluridine, and 2'-deoxyuridine

Fig. S3 shows examples of our 225 nm MPI mass spectra of uridine, 5-methyluridine, and 2'-deoxyuridine. As noted in previous works[12–14], it is interesting that a large proportion of the fragment ion masses in the uridine mass spectrum are consistent with previous mass spectra of ribose, either in terms of the detected ion itself or the neutral loss from uridine$^+$ required to produce the detected ion. By contrast, only a few relatively weak fragment ion channels are common in uridine mass spectra and in those of uracil or protonated uracil[15,16]. This carries several implications. Firstly, it suggests a greater propensity for the sugar part of the excited uridine ion to fragment than the base part. Secondly, the apparent detection of common ionization products from (ribose minus OH) in uridine and from isolated ribose suggest that OH loss may be the first step in a number of sequential dissociation pathways of

excited ribose$^+$. Thirdly, the value of the ribose mass spectrum for peak assignments in the uridine mass spectrum is a good example of the efficacy of the bottom-up approach for interpreting the radiation response of biomolecules.

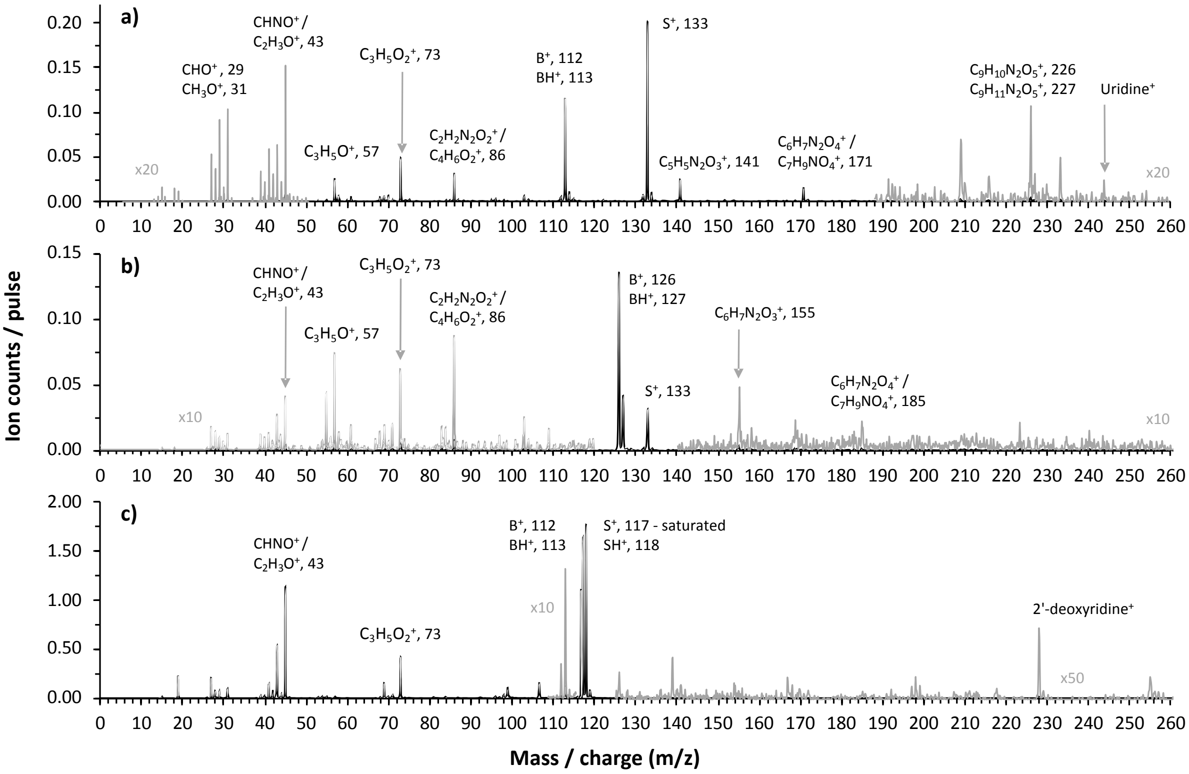

Fig. S3: 225 nm MPI mass spectra of (a) uridine, (b) 5-methyluridine, and (c) 2'-deoxyuridine. The desorption laser powers in these measurements were 0.41 W (foil temperature 145 ºC), 0.13 W (57 ºC), and 0.22 W (93 ºC), respectively. The most prominent fragment ion peaks of uridine are labelled with their previous assignments[13,14,17,18], whereas the peak assignments in panels (b) and (c) are proposed here based largely on an analogy with the uridine results. Note that the peak at about m/z 233 in panel (a) is due to delayed (µs-timescale) H_2O loss from excited uridine$^+$.

Unlike uridine, very few fragment ion assignments have been proposed previously for the peaks in 5-methyluridine[14] and 2'-deoxyuridine[12] mass spectra. The agreement of the three strongest peaks aside from B^+, BH^+, or S^+ (i.e. m/z 57, 73, and 86 – all also prominent peaks from ribose) in the uridine and 5-methyluridine mass spectra is a further indicator that fragment ion production from their common sugar part dominates. By contrast, it is intriguing that the mass spectra of deoxyribose in the literature[19] have relatively little in common with the 2'-deoxyuridine mass spectrum. For example, the strongest fragment ions from deoxyribose have m/z 44 and 59, both of which are absent in Fig. S3(c).

References

1 V. B. Delchev, *J. Mol. Model.*, 2010, **16**, 749–757.

2 R. So and S. Alavi, *J. Comput. Chem.*, 2007, **28**, 1776–1782.

3 I. Peña, C. Cabezas and J. L. Alonso, *Angew. Chemie Int. Ed.*, 2015, **54**, 2991–2994.

4 T. H. Dunning, *J. Chem. Phys.*, 1989, **90**, 1007–1023.

5 Y. Zhao, N. E. Schultz and D. G. Truhlar, *J. Chem. Phys.*, 2005, **123**, 161103.

6 D. Bakowies, *J. Phys. Chem. A*, 2013, **117**, 228–243.

7 L. A. Curtiss, K. Raghavachari, P. C. Redfern and J. A. Pople, *J. Chem. Phys.*, 2000, **112**, 7374–7383.

8 J. Kóňa and I. Tvaroška, *Chem. Pap.*, 2009, **63**, 598–607.

9 L. A. Burns, Á. Vázquez-Mayagoitia, B. G. Sumpter and C. D. Sherrill, *J. Chem. Phys.*, 2011, **134**, 084107.

10 N. Mardirossian and M. Head-Gordon, *Mol. Phys.*, 2017, **115**, 2315–2372.

11 M. J. Frisch, G. W. Trucks, H. B. Schlegel, G. E. Scuseria, M. A. Robb, J. R. Cheeseman, G. Scalmani, V. Barone, G. A. Petersson, H. Nakatsuji, X. Li, M. Caricato, A. Marenich, J. Bloino, B. G. Janesko, R. Gomperts, B. Mennucci, H. P. Hratchian, J. V. Ortiz, A. F. Izmaylov, J. L. Sonnenberg, D. Williams-Young, F. Ding, F. Lipparini, F. Egidi, J. Goings, B. Peng, A. Petrone, T. Henderson, D. Ranasinghe, V. G. Zakrzewski, J. Gao, N. Rega, G. Zheng, W. Liang, M. Hada, M. Ehara, K. Toyota, R. Fukuda, J. Hasegawa, M. Ishida, T. Nakajima, Y. Honda, O. Kitao, H. Nakai, T. Vreven, K. Throssell, J. J. A. Montgomery, J. E. Peralta, F. Ogliaro, M. Bearpark, J. J. Heyd, E. Brothers, K. N. Kudin, V. N. Staroverov, T. Keith, R. Kobayashi, J. Normand, K. Raghavachari, A. Rendell, J. C. Burant, S. S. Iyengar, J. Tomasi, M. Cossi, J. M. Millam, M. Klene, C. Adamo, R. Cammi, J. W. Ochterski, R. L. Martin, K. Morokuma, O. Farkas, J. B. Foresman and D. J. Fox, *Gaussian 09, Revision A.02*, Gaussian, Inc., Wallingford, 2016.

12 K. Biemann and J. A. McCloskey, *J. Am. Chem. Soc.*, 1962, **84**, 2005–2007.

13 S. Ptasińska, P. Candori, S. Denifl, S. Yoon, V. Grill, P. Scheier and T. D. Märk, *Chem. Phys. Lett.*, 2005, **409**, 270–276.

14 H. Levola, K. Kooser, E. Itälä and E. Kukk, *Int. J. Mass Spectrom.*, 2014, **370**, 96–100.

15 B. Barc, M. Ryszka, J. Spurrell, M. Dampc, P. Limão-Vieira, R. Parajuli, N. J. Mason and S. Eden, *J. Chem. Phys.*, 2013, **139**, 244311.

16 M. Pitzer, C. Ozga, C. Küstner-Wetekam, P. Reiß, A. Knie, A. Ehresmann, T. Jahnke, A. Giuliani and L. Nahon, *J. Phys. Chem. A*, 2019, **123**, 3551–3557.

17 P. O. P. Ts'o, *Basic Principles in Nucleic Acid Chemistry V1.*, Elsevier Science, 1974.

18 J. M. Rice and G. O. Dudek, *Biochem. Biophys. Res. Commun.*, 1969, **35**, 383–388.

19 National Institute of Standards and Technology (NIST), D-erythro-Pentose, 2-deoxy-, https://webbook.nist.gov/cgi/cbook.cgi?Name=deoxyribose&Units=SI&cMS=on#Mass-Spec, (accessed 12 January 2020).
